# Thin-film fixed-bed reactor for solar photocatalytic inactivation of *Aeromonas hydrophila*: influence of water quality

**DOI:** 10.1186/1471-2180-12-285

**Published:** 2012-11-29

**Authors:** Sadia J Khan, Robert H Reed, Mohammad G Rasul

**Affiliations:** 1 Centre for Plant and Water Sciences, Central Queensland University, Rockhampton, QLD, 4701, Australia

## Abstract

**Background:**

Controlling fish disease is one of the major concerns in contemporary aquaculture. The use of antibiotics or chemical disinfection cannot provide a healthy aquaculture system without residual effects. Water quality is also important in determining the success or failure of fish production. Several solar photocatalytic reactors have been used to treat drinking water or waste water without leaving chemical residues. This study has investigated the impact of several key aspects of water quality on the inactivation of the pathogenic bacterium *Aeromonas hydrophila* using a pilot-scale thin-film fixed-bed reactor (TFFBR) system.

**Results:**

The level of inactivation of *Aeromonas hydrophila* ATCC 35654 was determined using a TFFBR with a photocatalytic area of 0.47 m^2^ under the influence of various water quality variables (pH, conductivity, turbidity and colour) under high solar irradiance conditions (980–1100 W m^-2^), at a flow rate of 4.8 L h^-1^ through the reactor. Bacterial enumeration were obtained through conventional plate count using trypticase soy agar media, cultured in conventional aerobic conditions to detect healthy cells and under ROS-neutralised conditions to detect both healthy and sub-lethally injured (oxygen-sensitive) cells. The results showed that turbidity has a major influence on solar photocatalytic inactivation of *A. hydrophila*. Humic acids appear to decrease TiO_2_ effectiveness under full sunlight and reduce microbial inactivation. pH in the range 7–9 and salinity both have no major effect on the extent of photoinactivation or sub-lethal injury.

**Conclusions:**

This study demonstrates the effectiveness of the TFFBR in the inactivation of *Aeromonas hydrophila* under the influence of several water quality variables at high solar irradiance*,* providing an opportunity for the application of solar photocatalysis in aquaculture systems, as long as turbidity remains low.

## Background

Infectious diseases are one of the main constraints for the operation and expansion of the aquaculture industry. Aquaculture systems have been accused of causing many negative environmental impacts, including water pollution, destruction of mangrove forests, reduction in biodiversity, and salinisation of fresh water [1]. Chemical disinfection is an effective treatment for many types of pathogens, including viruses, bacteria, fungi and protozoan parasites [2]. Use of chlorination, ozone treatment or antibiotics generates potentially toxic by-products and can leave residues which not only affect fish condition but may also pose health risks to the human population [3].

Water quality is important in determining the success or failure of fish production in aquaculture systems [4], being one of the aspects that requires careful consideration [5]. Many physical and chemical water quality variables are involved in fish health [6]. These variables can be influenced by each other and by environmental and biological conditions [7]. Therefore, this study investigates the impact of several aspects of water quality on the inactivation of the fish pathogen *Aeromonas hydrophila* using a solar photocatalytic system under full sunlight. This study reports on the extent of oxygen-sensitive cell injury occurring in a thin-film fixed-bed reactor (TFFBR) with solar photocatalytic disinfection for several important water quality variables. This study also investigates and compares the levels of inactivation of *A. hydrophila* in filtered and unfiltered aquaculture pond water, to compare results using synthesised and natural waters.

To assess the viability of bacteria during solar disinfection, the conventional approach is to enumerate samples using plate counts on a suitable agar-based growth medium after exposure to sunlight using standard aerobic conditions (e.g. 24 h incubation at a suitable temperature). However, previous studies have demonstrated that ROS, derived mainly from aerobic respiration during the enumeration process, may inactivate sub lethally damaged bacteria and prevent their growth and enumeration under conventional aerobic incubation [8]. Tandon et al. also demonstrated that due to oxygen sensitivity, the enumeration of *Enterococcus faecalis* on selective media under aerobic condition is not sufficient to count injured bacteria [9]. Two main reasons for oxidative stress during enumeration are: (a) The presence of reactive components in the growth media which occurs either due to oxidation of nutrients during autoclaving or due to photo-oxidation of growth media components after autoclaving. (b) The cellular respiratory process of the growing bacteria on exposure to light. Due to cellular respiration “cell destruction” or “cell suicide” can occur in sub-lethally injured bacteria as their protective mechanism against oxidative stress are damaged and they are incapable of coping with the oxidative burst when they are rapidly growing on nutrient rich medium [10]. Such cells cannot be counted under standard aerobic conditions, but can be cultured under conditions where reactive oxygen species are neutralised (ROS-neutralised conditions), e.g., in growth medium supplemented with the peroxide scavenger sodium pyruvate and incubated under anaerobic conditions to prevent cellular respiration [8,11]. The significance of this was shown in our recent study using a solar photocatalytic reactor under different flow rates with low sunlight and high flow rates showing substantial sub-lethal injury of *A. hydrophila*[12].

pH is a major variable in aquaculture systems; it influences the survival and growth of fish in culture and affects the physiological condition of the end product [13]. Lower pH generally decreases the survival and reproductive maturity of fish, while high pH can cause toxic ammonia imbalance within an aquaculture system [6]. The acceptable pH range for water used in aquaculture production is typically from 6.5 to 9 [14]. In solar photocatalysis, pH is also one of the main variables affecting the process. At higher pH levels, TiO_2_ surfaces are negatively charged and repulse anionic compounds in water [15]. In contrast, at low pH the density of positively charged catalyst increases which can then form an electrostatic link with the negatively charged surfaces of bacteria, resulting in a higher rate of microbial photo-disinfection [16]. Herrera Melian and his co-workers showed higher bacterial inactivation at pH 5 than at pH 7.8 which is consistent with such proposals [17]. However, Rincon and Pulgarin did not find any differences in bacterial inactivation at pH 4–9 [18]. Consequently, this research investigated microbial inactivation at pH levels of 5, 7 and 9 using the TFFBR system, thereby covering the typical pH range of aquaculture systems [14].

The salinity of aquaculture pond water is an influential factor for fish survival and growth [13]. Selven and Philip stated that salinity can cause negative effects in aquaculture species, linked to the growth and production of toxins by pathogens [19]. They showed that salinity variation increased the virulence characteristics of *Vibrio harveyi* in aquaculture systems, reducing the immune response in the shrimp hosts and causing heavy mortality. Wang and Chen showed that 2.5% NaCl significantly increased the growth rate of *Photobacterium* spp. and that addition of the same amount of NaCl to the growth medium (Tripticase soy broth) also increased the virulence of this pathogen towards shrimps [20]. Seawater has a typical salinity of 3.5% [21]. Therefore, this study investigates the effect of salinity (with and without NaCl and sea salt at 3.5%) on the photocatalytic inactivation of *A.hydrophila* through the TFFBR system.

Imbalance in an aquaculture pond ecosystems can change the water transparency, due to additional suspended solids [22]. Turbidity refers to the indicator of cloudiness of a water sample due to the presence of suspended materials in the water that affect light transmission [23]; this can adversely affect fish health. Clays are one of the most common suspended materials present in aquatic systems [24]. Reduced phytoplankton production and increased growth of heterotrophic bacteria in aquatic systems have often been attributed to high clay turbidity levels and low light transmission levels [24,25]. In relation to solar disinfection, highly turbid water samples at 300 Nephelometric Turbidity Units (NTU), showed reduced microbial inactivation compared to less turbid or non-turbid samples, which may be due to shielding of microbes from sunlight by suspended materials [26]. In batch system solar disinfection, Joyce et al. found that, less than 1% of the total solar UV light would reach a depth of 2 cm in water with a turbidity of 200 NTU [27]. Therefore, EAWAG, the Swiss Federal Institute of Aquatic Sciences and Technology, recommended that water for solar disinfection batch systems need to be not more than 10 cm in depth and a turbidity level of not more than 30 NTU [28]. Rincon and Pulgarin observed that water turbidity negatively affected the photocatalytic inactivation of microbes and resulted in bacterial re-growth, supported by nutrients associated with the suspended particles [29]. They also stated that suspended particles absorb heat from sunlight and warm the water. Warmer water holds less oxygen and consequently affects microbial respiration and photocatalysis. Suspended particles also reduce light penetration capacity by their scattering effect. One recent research study used a batch sequential CPC reactor to eliminate water pathogens, with reduced exposure time and minimal user input compared to other systemsn [30]. However, most of the previous studies of turbidity in solar disinfection have been in batch reactors with TiO_2_ suspensions, rather than immobilized systems. Another recent investigation has developed a CFD (computational fluid dynamics) model for water disinfection through a CPC pilot-plant reactor [31]. However, no laboratory experiments were evaluated in that study to evaluate its practical efficiency. In contrast to batch reactors and CPC reactor systems, the TFFBR system evaluated in the present study is a single-pass system. The reaction on the surface of the TFFBR reactor is different, as water is not in a static condition. Therefore, this study reports for the first time the use of a single-pass flow-through TFFBR system to investigate the elimination of an aquaculture pathogen from water of different turbidities.

Suspended particles are not the only obstacle to light penetration; dissolved coloured materials also absorb sunlight of different wavelengths [32]. Natural organic matter is present in all surface water; humic acids are major component in natural waters which are brown in colour [28]. Humic acids are generally present in most surface waters at levels of up to 10 mg L^-1^[33]. These substances may act as photosensitisers under the influence of solar radiation [34,35]. This can cause oxidative damage to the cell membrane [36] and also may influence solar photocatalytic degradation via TiO_2_[37]. Doll and Frimmel showed a reduction in photocatalytic degradation of several chemicals (carbamazepine, clofibric acids and iomeprol) with 2 commercially available TiO_2_ preparations, in the presence of humic acids, with these substances competing for active sites and causing surface deactivation of the catalyst by adsorption [38]. In contrast, humic acids can also negatively affect solar disinfection by absorbing the radiation that passes through the water and this can decrease solar UV transmission [28] and reduce cell inactivation [34,36,37,39]. As humic acids have an attraction towards aqueous metal cations, they may be able to interact with microbial surfaces and protect them from solar UV disinfection [33]. Therefore, this study has investigated the use of the TFFBR system to disinfect aquaculture bacteria from water samples containing added humic acids.

Temperature and dissolved oxygen (DO) levels are two important variables in aquatic systems which also influence microbial solar disinfection [29,34,40]. However, in this study, the TFFBR is an open system where the temperature of the thin layer of the water cannot be readily controlled and will rapidly change during passage across the reactor plate in full sunlight. During the experiments, the ambient temperature of that day was noted and the reservoir water temperature was maintained. As experiments were performed through a 2 year time period in different seasons, further control of water temperature was not considered. Similarly, water samples used in this research were fully oxygenated due to a combination of (i) mixing [flow/agitation] and (ii) the thinness of the film of water across the photoreactor, at <0.3 mm. Photo-oxidation happens on the TFFBR reactor plate and while residual reactive oxygen species are present in the treated water, they are extremely short-lived with half-lives measured in milliseconds. Therefore, DO levels have not been considered further in this study.

## Methods

### Reactor

**Figure 1:**
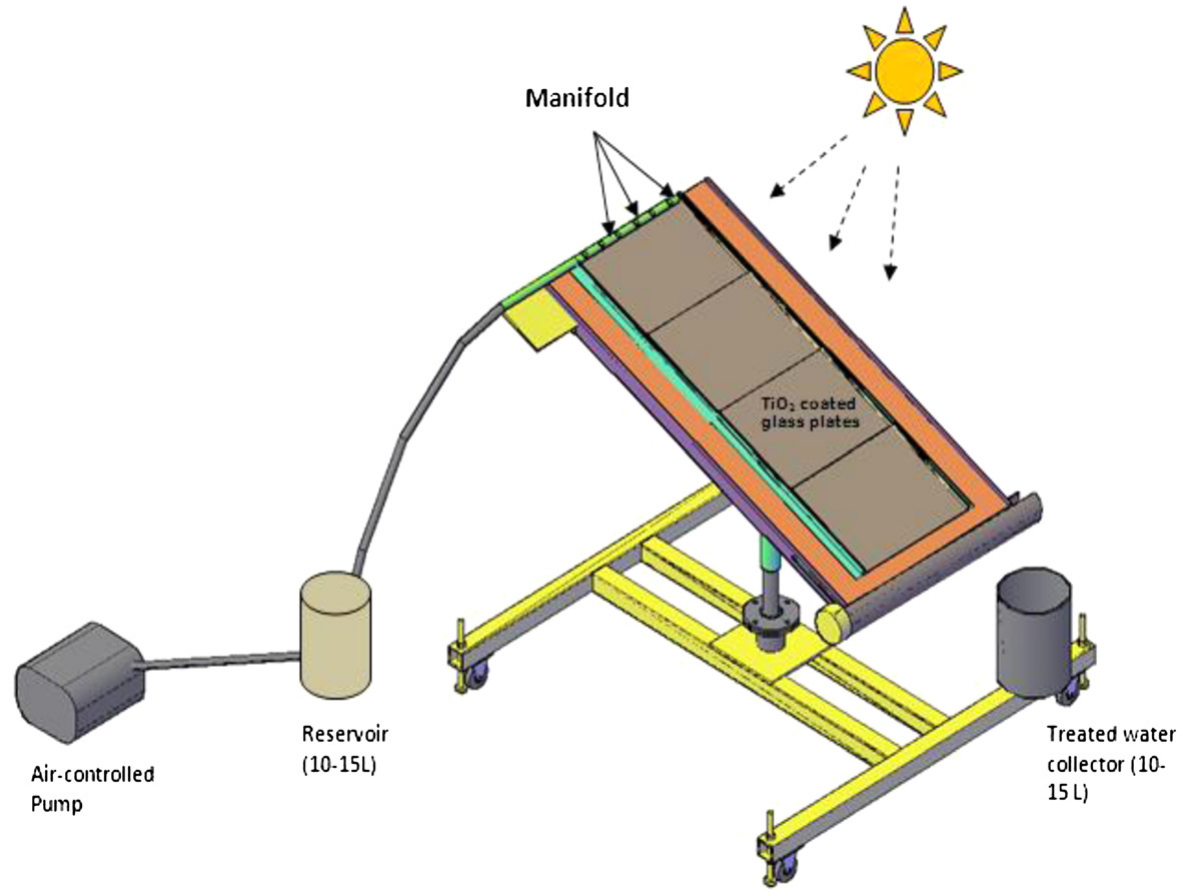
Schematic diagram and the thin-film fixed-bed reactor (TFFBR) used in this study

A pilot-scale solar photocatalytic thin-film fixed-bed reactor (TFFBR) system has been developed (Figure 1) and detailed by Khan et al. [12]. The overall experiment was set-up as a single-pass flow-through experiment. The reactor angle was maintained at 20^o^ to the horizontal and was kept as North-facing throughout the experiments to provide the best possible effect from natural sunlight, as reported in earlier studies [41]. The solar irradiance was measured in W/m^2^ using a Pyranometer (model SP1110, Skye instruments, UK) at the same angle as that of the reactor, giving readings during all experiments (full sunlight conditions) within the range 980–1100 W/m^2^. The illuminated surface area was 1.17 m in depth and 0.4 m in width (0.47 m^2^) and the irradiated volume was 200 mL with a residence time of 2.5 min at a flow rate of 4.8 L h^-1^ as in previous experiments [12]. The flow rate was 4.8 L h^-1^. The density of the TiO_2_ photocatalyst was 20.50 g m^-2^ and the photocatalyst layer was not covered during the experiments.

### Sources of water

Experiments on water quality variables were performed using autoclaved reverse osmosis (RO) treated water. Pond water experiments were performed by collecting aquaculture pond water from the Central Queensland University aquaculture pond system. To compare the pond water results sterile natural spring water (Satur8 Pty, Ltd, Australia) was also inoculated with *A. hydrophila* and investigated using the TFFBR system under similar experimental conditions.

For one set of experiments, pond water was filtered through 0.45 μm nitrocellulose Millipore filter paper (millipore coporation, Bellerica, MA, 01821) by a vacuum pressure mediated filter apparatus (Nalgene^R^, Thermo-Fisher Scientific Pty, Ltd, Australia). Then the filtered pond water was autoclaved again before use. In another set of experiments, pond water was not filtered, only autoclaved.

### Bacterial culture and experimental procedure

*Aeromonas hydrophila* ATCC 35654 was purchased from Oxoid, Australia. This was maintained by repeated sub-culture on trypticase soy agar (TSA) (Oxoid, Australia) at 25°C. Culture maintenance, experimental set up, and experimental procedure were as described previously [12]. For lab enumeration, each sample was processed by serial decimal dilution to cover the range 10^0^-10^-2^. Then three aliquots of 20 μL of each dilution were plated by the droplet spread plate technique [9] on TSA with or without 0.05% w/v sodium pyruvate and incubated at 25°C for 48 h. Plates without sodium pyruvate were incubated in a conventional aerobic incubator (Cotherm, Biocell 1000, Thermo Fisher Scientific Ltd. Australia), to provide counts of healthy bacteria. Aerobic and RO-neutralised enumeration techniques were detailed in our earlier study [12]. This study considered only one flow rate, 4.8 L h^-1^ and high solar irradiance conditions 980–1100 W m^-2^, as previous studies demonstrated that this combination of low flow rate and high solar irradiance condition provided the most effective condition for microbial inactivation [12]. All experiments were repeated 3 times on 3 different days. For each experiment 3 different water samples were collected and enumerated every 10 min within a single 30 min period. Therefore on 3 different days, the sample size was 3 × 3=9 distinct samples/counts. To provide a measure of the inactivation that occurred during solar photocatalysis, the log-transformed count of sunlight-treated water at each time point were subtracted from the log-transformed count of untreated water (dark control) to provide an overall value for log inactivation. All data shows are mean values across all experiments. The individual results for all 9 values have been used to calculate means and 95% confidence limits.

### pH experiments

For these experiments, 3 sets of RO water samples were prepared and pH levels were adjusted using diluted NaOH and HCl to achieve pH conditions of 5, 7 and 9. Each day, 3 batches of experiments were performed with 3 different pH conditions under full sunlight at 4.8 L h^-1^ flow rate. To investigate the extent of pH levels for survival of *A. hydrophila* another experiment was performed in dark with the pH conditions of 5, 7 and 9. RO samples with pH levels 5, 7 and 9 were prepared with similar initial counts of *A. hydrophila* to those of photocatalytic experiments and these were then kept in darkness for 9 hours, with sampling at 0 min and 9 hour. Each sample was serially diluted and enumerated.

### Salinity experiments

For these experiments, reverse osmosis (RO) treated water was used so that no additional salts would be present. Three sets of water samples were used for the salinity experiments. (1) RO water containing 3.50% w/v NaCl (2) RO water containing 3.50% w/v sea salt Sea salt (AnalaR, chemicals Ltd, BDH, UK) and (3) RO water with 0% added salt (control) were prepared and autoclaved before use. A conductivity meter (Thermo Orion 4 star, Thermo-fisher Pty. Ltd, Victoria, Australia) was used to measure saline conductivity in μS/cm.

### Water turbidity experiments

A kaolin suspension was prepared according to Wilson and Andrew [32]. Ten grams of kaolin powder (Thermo-Fisher Scientific, Australia) was added to 2 L of RO water, stirred for 1 h and kept overnight to settle. Then the supernatant containing any dissolved contaminants was discarded and the remaining portion was diluted into a 10 L volume of RO water. The turbidity measurement of the resulting suspension was 810 NTU. Different volumes of this kaolin suspension were taken and added to RO water to produce water with turbidity of 0, 23, 58 and 108 NTU, which were then autoclaved before use. Each day 4 sets of these different turbid waters were used to find the effect of different turbidity levels on inactivation of *A. hydrophila*. Experiments were repeated 3 times on 3 different days.

### Humic acid experiments

In order to an prepare experimental solution of RO water with humic acid, a stock solution was prepared with a mixture of 500 mg of technical grade humic acid, sodium salt (Sigma-Aldrich, USA) and 50 mL ethanol (100%). As up to 10 mg L^-1^ humic acids are present in surface waters [33], the test concentration of humic acid required for each experiment was selected as 10 mg L^-1^. Consequently, 6 mL of stock solution was added to 5994 mL of RO water for each experiment. For control experiments 6 mL of 100% ethanol was added to 5994 mL of RO water. Each water sample was autoclaved before use. Each experiment was repeated 3 times on 3 different days.

### Aquaculture pond water experiments

Two sets of pond water (filtered and unfiltered) were used for experiments. For each pond water experiment, pH, salinity, and turbidity were measured, before and after each experiment were performed. In addition nine turbidity measurements in NTU were taken monthly from Dec, 2010- Oct 2011 to establish the effect of season on turbidity levels. Pond water experimental results were compared with equivalent experiments using spring water (Satur8 Pty Ltd, Australia). Autoclaving was the only practical option for sterilisation of aquaculture water, due to the high level of turbidity and suspended particulates, which meant that membrane filtration was not an option.

## Results

### Effect of pH

**Figure 2:**
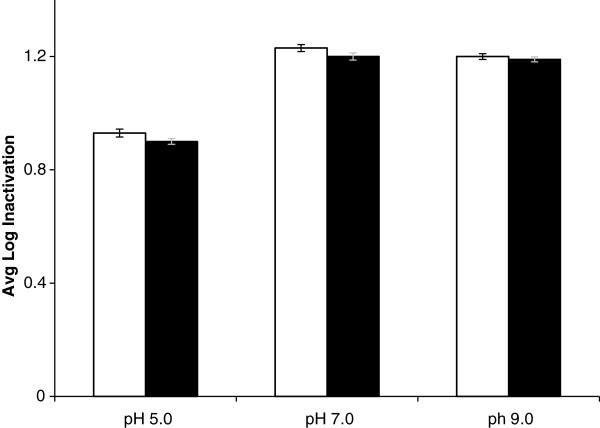
**Effect of pH on solar photocatalytic inactivation of *Aeromonas hydrophila *ATCC 35654.** TFFBR experiments were performed at average value of global irradiance of 1034 W m^-2^, at a flow rate of 4.8 L h^-1^. Enumeration was carried out under aerobic conditions (unshaded bars) and ROS-neutralised conditions (shaded bars)

Figure 2 shows the effect of pH on average log inactivation of *A.hydrophila* ATCC 35654 at high solar irradiance (980–1100 W m^-2^) at a flow rate of 4.8 L h^-1^. The log inactivation represents the difference in log counts between inflow and outflow of the TFFBR system. pH 7.0 and 9.0 both showed a slightly higher average log inactivation than at pH 5.0 with an average log inactivation of approximately 1.2 at pH 7.0 and 9.0 where the average initial level of *Aeromonas hydrophila* was 5.1 Log CFU mL^-1^ and the average final count was 3.9 Log CFU mL^-1^. On the other hand, for pH 5 the average log inactivation was less, at 0.9, where the average initial count was 4.9 Log CFU mL^-1^ and the final average counts was 4.0 Log CFU mL^-1^. Overall, the results suggest only a small effect of pH on photoinactivation, irrespective of whether the sample was counted under aerobic or ROS-neutralised conditions.

However, all pH 5.0 experiments showed a reduced initial count prior to exposure to the TFFBR, even though the volume of the cultured bacteria inoculated into the water was the same in every pH experiment. Therefore, a question arose as to the reason of this difference.

**Figure 3:**
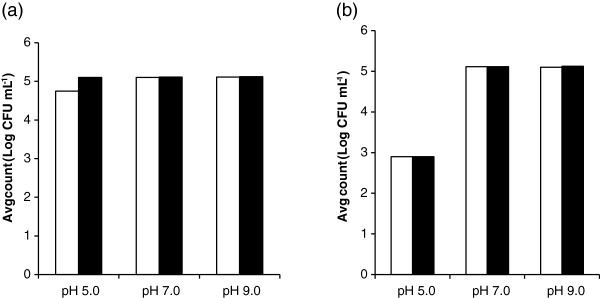
**Average survival counts of *A. hydrophila *following storage at different pHs.** Enumeration was carried out after storage for 0 min (**a**) and 9 hr (**b**), under aerobic (unshaded bars) and ROS neutralised (shaded bars) conditions for water sample kept in darkness for 9 hr at pH 5.0, 7.0 and 9.0

In Figure 3, pH 7.0 and 9.0 showed similar initial counts of 5.1 log CFU mL^-1^ for *A. hydrophila* in both aerobic and ROS-neutralised condition. But at pH 5 this initial count was log 4.75 log CFU mL^-1^ under aerobic condition, where under ROS-neutralised condition it was higher, at 5.1 log CFU mL^-1^. This points to some sub-lethal injury on exposure of this organism to water at pH 5.0. After 9 hr, pH 7.0 and 9.0 samples showed the average counts of bacteria remained at 5.1 log CFU mL^-1^, enumerated under both aerobic and ROS-neutralised conditions. However, for pH 5.0 it showed a large reduction in the counts compared to those at 0 min, at approximately 2.9 log CFU mL^-1^ in both aerobic and ROS-neutralised conditions. This demonstrates that storage of *A. hydrophila* at pH 5 causes a reduction in the count of bacteria and the similar counts under aerobic and ROS-neutralised conditions point to irreversible inactivation.

### Effect of salinity

**Figure 4:**
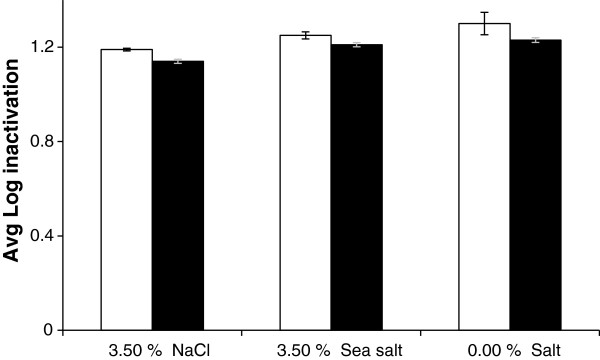
**Effect of different saline conditions on the inactivation of *Aeromonas hydrophila *ATCC 35654.** Experiments were carried out using the TFFBR system under an average value of global irradiance of 1022 W m^-2^at 4.8 L h^-1^. Cell enumeration was done under aerobic (unshaded bars) and ROS neutralised (shaded bars) conditions

Figure 4 shows the effect of different saline condition (3.50% NaCl, 3.50% sea salt and 0.0% salt) on average inactivation of *A. hydrophila* ATCC 35654. All 3 conditions showed a similar degree of inactivation. Overall, it is clear that variation in salinity conditions with NaCl or sea-salt at 3.50% had no substantial effect on solar photocatalysis in the TFFBR at high sunlight and low flow rate conditions. In these experiments no sign of salt crystallisation was observed due to evaporation on the TFFBR plate.

### Effect of turbidity

**Figure 5:**
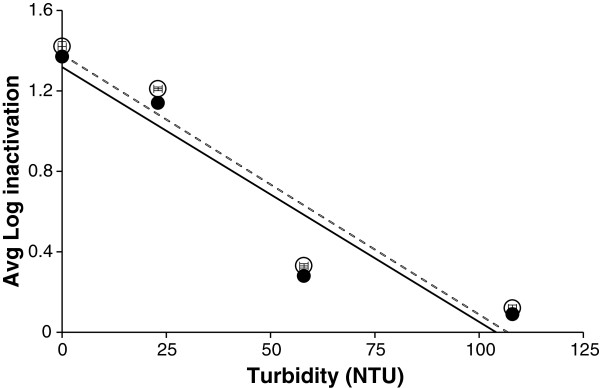
**Effect of turbidity on the inactivation of *Aeromonas hydrophila *ATCC 35654.** Experiments were carried out using the TFFBR under an average value of global irradiance of 1033 W m^-2^ at low flow rate (4.8 L h^-1^). Enumeration was performed under aerobic (open circles) and anaerobic ROS neutralised (closed circles) conditions

In order to investigate the effect of water of different turbidity, Figure 5 was plotted to show the log inactivation counts against turbidity where the initial count was 5.1 log CFU mL^-1^. It showed that with 0 NTU turbid water sample, 1.3 log inactivation was observed for both aerobic and ROS-neutralised conditions. The extent of inactivation gradually decreased with increasing levels of turbidity e.g. water samples with 23 NTU, 58 NTU and 108 NTU showed an average log inactivation of 1, 0.28 and 0.09, respectively under both aerobic and ROS-neutralised conditions. Under high solar irradiance condition the data also show that inactivation was not accompanied by sub-lethal injury across this turbidity range. It is clear that less turbid water samples favour more microbial inactivation.

Linear regression trend lines were plotted with both sets of data obtained from the counts under aerobic and ROS-neutralised conditions. Both conditions predicted best fit lines with positive intercept close to 1.3 with similar regression coefficient values of 0.89 (Table 1). As the regression coffients are close to 1, they show a strong fit of the data to the linear trend line where microbial inactivation decreases as the water turbidity increases.

**Table 1:** **Linear regression analysis for inactivation of *A.hydrophila *ATCC 35654 under different turbidity level from 0–108 NTU based on the data shown in Figure**4

**Enumeration condition**	**Linear regression equation**	**R**^**2**^**values**
Aerobic	Y= − 0.010X + 1.318	0.89
ROS-neutralised	Y= − 0.012X + 1.380	0.89

### Effect of humic acid

**Figure 6:**
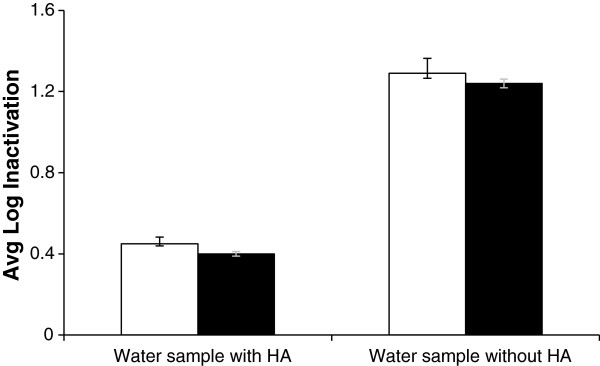
**Effect of humic acid (HA) on the inactivation of *Aeromonas hydrophila *ATCC 35654.** Experiments were carried out using the TFFBR under an average value of global irradiance of 1037 W m^-2^at low flow rate 4.8 L h^-1^. Enumeration was performed under aerobic (unshaded bars) and ROS neutralised (shaded bars) conditions

Figure 6 shows the log inactivation of *A. hydrophila* for water samples with or without humic acid at 10 mg L^-1^ through the TFFBR system. Water samples with humic acid showed almost 0. 4 log inactivation in both aerobic and ROS-neutralised condition. On the other hand water samples without humic acid showed almost 1.3 log inactivation in both conditions. This is close to a ten fold difference in the actual level of inactivation between these samples. Both water samples had initial counts of 1.4 × 10^5^ CFU mL^-1^ whereas without humic acids this dropped to 1.0 × 10^4^ CFU mL^-1^ after TFFBR while with humic acids this stayed high at 5.0 × 10^4^ CFU mL^-1^ after TFFBR. Under full sunlight condition in the TFFBR, there was negligible cell injury observed, since similar counts were obtained under aerobic and ROS-neutralised conditions. It is clear that a humic acid content of 10 mg L^-1^ has a major negative effect on solar photocatalysis at high sunlight and low flow rate conditions.

### Comparison of inactivation of *A. hydrophila* in pond water and spring water

**Figure 7:**
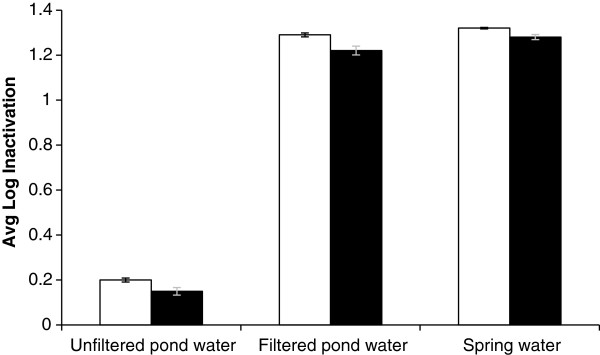
**Comparison of log inactivation of *A. hydrophila *ATCC 35654 inoculated in pond water (filtered, un-filtered) and spring water.** Experiments were carried out using the TFFBR under an average value of global irradiance of 1021 W m^-2^ at low flow rate 4.8 L h^-1^. Enumeration was carried out at under aerobic (unshaded bars) and ROS neutralised (shaded bars) conditions

Figure 7 shows the differences in the inactivation levels of *A. hydrophila* inoculated into aquaculture pond waters (filtered and unfiltered) and spring water and then run across the TFFBR plate under high solar irradiance conditions. Filtered pond water and spring water showed a similar level of *A. hydrophila* inactivation within a range of 1.22 - 1.32 log inactivation under both aerobic and ROS-neutralised conditions, where the initial count was 5.1 log CFU mL^-1^. On the other hand, with the same experimental conditions, unfiltered pond water showed a log inactivation of 0.2 under aerobic condition and 0.15 log CFU mL^-1^ under ROS-neutralised condition. During the experiments, several water quality variables (pH, salinity conductivity and turbidity levels) were measured before and after treating the water samples through the TFFBR (Table 2).

**Table 2:** **Experimental conditions of different variables while conducting the inactivation of *A .hydrophila *through TFFBR**

**Experiment No.**	**Parameters**	**Filtered pond water**		**Un-filtered pond water**		**Spring water**	
		**Before treatment**	**After treatment**	**Before treatment**	**After treatment**	**Before treatment**	**After treatment**
1	pH	8.7	8.8	8.8	8.69	8.03	8.08
	Conductivity (μS/cm)	321	370	269	301	0	0
	Turbidity (NTU)	1	1	69	71	0	0
2	pH	8.9	9	8.89	9.01	8.1	8.07
	Conductivity (μS/cm)	200	233	289	313	0	0
	Turbidity (NTU)	2	1	72	70	0	0
3	pH	7.96	8	8.78	8.8	7.9	8.01
	Conductivity (μS/cm)	188	205	197	214	0	0
	Turbidity (NTU)	3	2	51	50	0	0

Table 2 shows that there was no major change in pH levels during the experiments for each water sample. Salinity (conductivity) levels were slightly higher with the pond waters (filtered or un-filtered) once they had passed across the TFFBR. This is logical since, due to the high sunlight a small amount of evaporation will occur and salt concentration will increase. However, the extent of water evaporation was so small that no visible salt crystallisation was observed on the TFFBR plate itself. In the spring water sample, the conductivity level was 0 μS/cm in every experiment while in pond waters the values were within a range of 188–370 μS/cm, using either filtered or unfiltered pond water. However, it is worth mentioning that filtered pond water and spring water showed a similar range of log inactivation of 1.2, which is a ten-fold higher level of inactivation than that of the un-filtered pond water. Even though, there was more than 200 μS/cm difference in the salinity levels among the spring water and pond water, there was no significant difference in microbial inactivation observed between them. Such similar findings were also evident from Figure 4, where variations in salinity using NaCl or sea-salt caused no major effect on solar photocatalysis through the TFFBR system.

Figure 7 showed a difference of almost 1 log inactivation between the filtered and un-filtered pond water. Since pH and salinity showed no major effect to support this difference in individual experiments (Figures 2 and 4), it seems reasonable to propose that the other measured variable, turbidity, is likely to have a major role. From Table 2, every experiment with unfiltered pond water showed a turbidity level at or above 50, whereas the turbidity levels for spring water and filtered pond water were only 0 and 1–3, respectively. Experimental results from Figure 4 also showed that highly turbid water samples have a negative effect on solar photocatalysis. So, it is logical that, the less turbid filtered pond water will result in greater microbial photocatalytic inactivation through the TFFBR system compared to unfiltered pond water of high turbidity and the degree of change in log inactivation resulting from filtration and consequent decrease in turbidity is consistent with the data shown in Figure 5.

**Figure 8:**
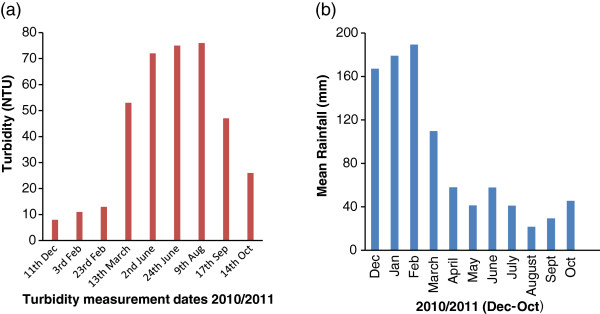
**(a) Turbidity measurements of un-filtered pond water of the CQUniversity aquaculture system, starting from Dec, 2010 –Oct 2011. **(b) Mean rainfall (mm) in Dec, 2010/Oct, 2011- data obtained from Bureau of Meteorology, Government of Australia

The pond water experiments were performed during the winter season to avoid rain interruptions that happen frequently during summer season. Pond water turbidity levels vary due to various weather conditions in winter, summer and in rainy seasons. Therefore, the turbidity measure of unfiltered pond water was measured monthly, starting from Dec, 2010 to Oct 2011 and plotted in Figure 8.

In Central Queensland, spring and summer seasons (November, 2010 to March, 2011) are accompanied by heavy rainfall. Figure 8 (b) shows the mean rainfall of each month from Dec, 2010 to Oct 2011. Figure 8 (a) showed the turbidity levels of the pond water varied over the range 8–76 NTU during the period Dec, 2010- Oct, 2011. Comparing the data from Figure 8 (a) and (b), it can be determined that the turbidity levels were lowest (8–16 NTU) during the summer period which is linked to heavy rainfall conditions, with a high mean rainfall of 180 mm in Jan, 2011. During winter minimal rainfall was observed with a low in August of 22 mm of around rain when the turbidity level was high, at 76 NTU. So it is logical to interpret from these observations that the summer season will provide better microbial photocatalytic inactivation over the winter period due to a combination of high sunlight and lower turbidity.

## Discussion

This study has showed that there was a relatively small effect of pH 7.0 and pH 9.0 on microbial inactivation. pH 5.0 showed a different result in Figure 2 with a lower initial counts. As, the acceptable range of a healthy aquaculture system is within the pH range of 6.5 to 9 [14], the findings from Figure 2 at pH levels of 7.0 and 9.0 demonstrates that there is no major pH effect against *A. hydrophila* inactivation over this pH range. Rincon and Pulgarin [18] suggested that modifications of water pH between 4 and 9 had no effect on photocatalytic batch culture solar disinfection of *E. coli*. However, the catalyst would be more negatively charged at high pH and the result is therefore not as might be predicted on the basis of charge alone, indicating that other factors must be involved. To clarify the reduced initial count at pH 5.0 in Figure 2, a longer-term storage experiments was performed over 9 h (Figure 3) to find out the survival capacity of *A. hydrophila* at pH levels of 5.0, 7.0 and 9.0. This illustrated that in darkness, pH 5.0 negatively affected the survival of *A. hydrophila*. Some previous aquaculture studies provided evidences that low pH levels are not suitable for growth and survival of fish species [6,13,42]. Therefore, the result at pH 5.0 is of less direct relevance to aquaculture systems, since this is not within the usual range of operations.

Fresh water ponds, tanks and cages provided 60% of the total aquaculture production of the world in 2008 [43]. Similarly, coastal ponds and tanks also produce fish, molluscs, crustaceans etc. In warm regions, prawns and shrimps mainly dominate the world’s total aquaculture production, 58% of which comes from brackish water supply [44]. Marine-based aquaculture has also expanded to produce sea fish, mollusc and seaweeds and the total value from this was almost US$ 20 billion in 2008 [44]. There are few studies on the effect of salinity on aquaculture systems, which mainly focus on fish mortality and the influence of salinity increase on the susceptibility of fish to certain pathogens [19,20]. This current study is the first study to reveal the possibility of application of TFFBR to aquaculture systems with saline waters. The findings of this research, clearly demonstrates that there is no substantial effect of salinity on *A. hydrophila* inactivation at the level of salt observed in sea water. So, it is evident that this TFFBR technique may be applicable to aquaculture systems containing fresh water, brackish water or marine water.

The effect of turbidity was also investigated in this study by flowing contaminated RO water with different turbidity levels across the TFFBR under high solar irradiance conditions. The findings of this study confirmed a trend show by Hirtle [45], which was that the presence of inorganic particles (kaolin) decreased the efficiency of solar disinfection treatment. Hirtle explored the pre-treatment for solar disinfection by using filters in 2 litre PET water bottles having a hole at the bottom and using a peristaltic pump to flow the turbid water samples (kaolin-containing water with different turbidity levels) contaminated with *E. coli* under total sunlight condition of 322–1068 W m^-2^[45]. In contrast, Wilson demonstrated that there was no obvious trend between the presence of inorganic kaolin particles across a range of turbidity levels in water samples from 0–200 NTU and *E .coli* log reduction under various sunlight irradiances for 7 h [28]. In another recent research study by Fontán-Sainz et al. ([2012]) using a solar CPC reactor, there was a significant loss of efficiency in the inactivation of *Crytosporidium parvum* oocysts under full sunlight conditions when the water turbidity increased from 5 to 30 NTU [46]. The study of Wilson [28] used a batch culture reactor whereas Fontán-Sainz et al. [46] used an uncatalysed solar system for their disinfection treatment and these are both different methods compared to the present study using the continuous flow TFFBR system. The present study used a different TiO_2_ reactor (immobilised form) and found a similar pattern of decreased microbial inactivation with increased turbidity. Chen et al. ([2010]) used kaolin in a lab-scale fixed TiO_2_ photocatalytic experiment to examine the microbial removal efficiency through a reactor [47]. In their study, TiO_2_ was synthesized by the sol–gel technique and they deposited 100 μl of phosphate buffer saline (PBS) containing bacteria on to a TiO_2_ coated glass plates which in turn was exposed to UV irradiation for 30 min. The authors demonstrated that a high concentration of kaolin particles (water with 100 NTU) was required to reduce the solar photocatlytic inactivation of *E. coli* and *S. aureus* in their system*.* Rincon and Pulgarin showed in their batch culture photocatalytic system that microbial inactivation decreased when the water turbidity increased from 27 to 84 NTU [29]. The present study treated a contaminated water sample in a single-pass reactor, receiving only a few minutes of full sunlight on the TFFBR plate. Under these conditions microbial inactivation decreases with the increasing turbidity levels in water. The present study showed a greater level of inactivation of *A. hydrophila* when the turbidity levels were less than 30 NTU, which agrees with the level recommended for the application of solar/solar photocatalytic disinfection by EAWAG [29]. Therefore, this study shows that the TFFBR system is efficient enough to eliminate aquaculture pathogens from less turbid water samples. As the difference in inactivation counts observed between the aerobic and ROS-neutralised condition were negligible, this can be interpreted to show that TFFBR under high solar irradiance conditions gives complete inactivation of microorganism with minimal sign of cell injury (ROS-sensitivity).

The addition of humic acid to water had a considerable effect on microbial inactivation during TFFBR treatment. After a single pass, the amount of disinfection was inversely related to the humic acid content of the water under s. This result agrees with Wilson [28], who used batch reactors under sunlight for 7 h to disinfect *E.coli* with water samples over a range of humic acid concentration 0–32 mg L^-1^. Wilson showed only 0.3 log reduction when the humic acid concentration was 32 mg L^-1^. On the other hand, it was 5.8 log reductions when humic acid content was 0 mg L^-1^. The present study showed around 0.4 log reduction of *A. hydrophila* with a humic acid content of 10 mg L^-1^. While the reactor and experimental features used in this present study were very different from Wilson [28] but the findings were similar. Since humic acid can also act as a photosensitiser [35], it might have facilitated the photo-oxidation process with more cell inactivation, but this was not the observed outcome. As humic acids are constituents of many natural water and affect microbial inactivation, for future researchers it could be useful to investigate long term chemical actinometry and related microbial studies.

In aquaculture pond water experiments, only turbidity was found to be an influential factor affecting microbial inactivation while treating filtered and un-filtered pond water. Based on single factor experiments (Figures 2 and 4) it can be proposed that pH and salinity levels will not substantially affect microbial inactivation in pond water treatment. Figure 7 illustrated that inactivation of *A. hydrophila* in unfiltered water was 1 log higher than the filtered water sample. Filtered pond water and spring water samples provided similar level of microbial inactivation, so it is clear that any colour components in the pond water sample were not an obstacle to microbial inactivation.

The current research was conducted a single pass reactor which caused a reduction in the log count of *Aeromonas hydrophila* of 1–1.4-fold as a result of this single pass. Our earlier study [12] also showed TiO_2_ coated glass plate was more effective than un-coated glass plate TFFBR in microbial inactivation. For an aquaculture system, the system would operate in recirculation mode, with a continuous flowing TFFBR reactor treating an aquaculture pond under high sunlight condition. This should help to maintain the population of pathogens such as *Aeromonas hydrophila* population below the infective dose, thereby preventing the establishment of an infection. The minimum infectious dose of *A. hydrophila* varies from strain to strain - for example, a dose of 10^5^ cfu of *A. hydrophila* AL0179 per fish (Nile tilapia) has been shown to cause 20% mortality [48]. As a whole, the use of TFFBR in aquaculture systems is a new technology that may be applicable to fresh water, brackish water or marine systems. From Figure 8, it was clearly seen that during the summer season the turbidity of aquaculture pond water was lower while in the winter it was high because of the weather conditions. Therefore, the TFFBR system will be more useful for treating aquaculture pathogens such as *A. hydrophila* when the water turbidity is lowest in the summer season, being likely to be less effective in winter due to a combination of higher turbidity and lower solar irradiance. Above all, to get microbial inactivation in this study, both bacterial enumeration techniques (aerobic and ROS-neutralised) were important as ROS-neutralised conditions shows the number of damaged (ROS-sensitive) cells under similar experimental conditions.

## Conclusion

The results clearly show that turbidity has a significant influence on solar photocatalytic inactivation of *A. hydrophila* using the TFFBR system with synthetic and natural waters. Humic acid added to water samples also caused a noticeable reduction in microbial inactivation. pH 5 decreases inactivation while salinity (0.00-3.50%) had no major effect on *A. hydrophila* inactivation. Finally, the observation that the turbidity of aquaculture pond water had a substantial effect on microbial inactivation is likely to affect the operation of aquaculture systems, especially in winter months.

## Competing interests

All authors confirm that there is no competing interest.

## Authors’ contributions

The project was designed by SK; RR and MR. All experiments were performed by SK under supervision of RR. The paper was co-drafted by SK and RR. All authors approved the final version of the manuscript.
